# Correction: Self-esteem in crisis: Psychosocial adaptation and masculine identity among Chinese men with azoospermia

**DOI:** 10.1371/journal.pone.0357353

**Published:** 2026-09-01

**Authors:** The PLOS One Staff

## Notice of Republication

This article was republished on March 16, 2026, to correct an error in the title: Self-esteem was erroneously displayed as “estee m”. The publisher apologizes for the error. Please download this article again to view the correct version. The originally published, uncorrected article and the republished, corrected article are provided here for reference.
